# Personal

**Published:** 1917-07

**Authors:** 


### PERSONAL

---

Dr. C. J. Patterson, Physician in Charge of the Marshall Sanitarium of Troy, attended the banquet of the Buffalo Academy of Medicine, June 13. He was formerly a Buffalonian.

---

Dr. W. D. Preston of Attica, who enlisted in the Medical Corps at the time of the Spanish-American War, has been ordered to the medical training camp at Ft. Benjamin Harrison, Ind.

Dr. Obert Pollock of San Diego, called on us last month, on his way home from the A. M. A. meeting.

